# Endocrine-Disrupting Chemicals and Hypertension: Mechanisms and Clinical Implications

**DOI:** 10.3390/jcdd13090462

**Published:** 2026-09-14

**Authors:** Müge Keskin, Gökhan Rıza Baykal, Arzu Or Koca, İlhan Yetkin, Mustafa Cesur

**Affiliations:** 1 Department of Endocrinology and Metabolism, Dr. Abdurrahman Yurtaslan Ankara Oncology Training and Research Hospital, 06200 Ankara, Türkiye; muge.keskin@sbu.edu.tr (M.K.);; 2 Department of Endocrinology and Metabolism, Mardin Training and Research Hospital, 47420 Mardin, Türkiye; 3 Department of Endocrinology and Metabolic Diseases, Faculty of Medicine Hospital, Gazi University, 06500 Ankara, Türkiye; 4 Department of Endocrinology and Metabolism, Ankara Güven Hospital, 06540 Ankara, Türkiye

**Keywords:** endocrine-disrupting chemicals, hypertension, PFAS, bisphenol A, preeclampsia, endothelial dysfunction, oxidative stress

## Abstract

Hypertension remains one of the leading causes of global morbidity and mortality and is the leading modifiable risk factor for cardiovascular disease worldwide. The inability of conventional risk factors alone to fully explain the global prevalence of hypertension, together with accumulating evidence implicating endocrine-disrupting chemicals (EDCs) in the pathogenesis of hypertension, has led to growing scientific interest in this field over recent years. EDCs are exogenous environmental chemicals capable of disrupting hormone synthesis, transport, metabolism, and signaling pathways, thereby adversely affecting vascular and metabolic homeostasis. In particular, per- and polyfluoroalkyl substances (PFASs), bisphenol A, phthalates, PCBs, heavy metals, and pesticides have been associated with hypertension and may influence blood pressure regulation through multiple interconnected mechanisms, including oxidative stress, endothelial dysfunction, inflammation, epigenetic modifications, and activation of the renin–angiotensin–aldosterone system. Moreover, prenatal exposure to EDCs has been associated with hypertensive disorders of pregnancy, including gestational hypertension and preeclampsia, with potential implications for long-term maternal cardiovascular health. This review provides a comprehensive overview of the experimental and epidemiological evidence linking endocrine-disrupting chemicals to hypertension, with particular emphasis on the underlying molecular mechanisms, emerging clinical evidence, and future research directions.

## 1. Definition and Global Burden of Hypertension

Hypertension is one of the leading modifiable risk factors for cardiovascular disease, affecting approximately 1.3 billion adults worldwide, while adequate blood pressure control is achieved in only 20% of patients despite substantial advances in antihypertensive treatment [1,2]. According to a comprehensive global analysis by the Non-Communicable Diseases Risk Factor Collaboration (NCD-RisC) study, the number of individuals living with hypertension nearly doubled between 1990 and 2019, with the greatest increase observed in low- and middle-income countries. Although treatment and control rates have improved over time, these advances have remained largely confined to high-income countries [3].

Nevertheless, the pathogenesis and global burden of hypertension cannot be fully explained by traditional cardiovascular risk factors alone. Increasing attention has therefore focused on environmental exposures as potentially modifiable determinants of blood pressure and cardiovascular risk. Among these exposures, endocrine-disrupting chemicals (EDCs) are of particular interest because, unlike many traditional risk factors, they are widely distributed in the environment and may influence blood pressure regulation through multiple biological systems simultaneously. EDCs can interfere with hormonal signaling, vascular homeostasis, renal sodium regulation, adrenal steroidogenesis, and metabolic regulation, while also promoting oxidative stress and inflammatory responses. Importantly, exposure may occur throughout the life course, beginning during fetal development, when disruption of endocrine and epigenetic programming may have persistent cardiovascular consequences. Furthermore, individuals are typically exposed to complex mixtures of environmental chemicals rather than to isolated compounds, potentially resulting in additive or interacting biological effects. These characteristics provide a rationale for considering EDC exposure as an emerging environmental component of hypertension risk and for evaluating its contribution alongside established genetic, metabolic, behavioral, and socioeconomic determinants of blood pressure [4,5].

## 2. Literature Search Strategy

This article was designed as a narrative review. The relevant literature was identified through searches of PubMed/MEDLINE, Scopus, and Web of Science using combinations of terms including “endocrine-disrupting chemicals,” “hypertension,” “blood pressure,” “PFAS,” “bisphenol A,” “phthalates,” “polychlorinated biphenyls,” “pesticides,” “heavy metals,” “endothelial dysfunction,” “oxidative stress,” “renin–angiotensin–aldosterone system,” and “pregnancy.” Priority was given to recent original studies, systematic reviews, meta-analyses, scientific statements, and mechanistic studies directly relevant to EDC exposure and blood pressure regulation. Additional references were identified from the bibliographies of selected articles. Because this was a narrative rather than systematic review, no formal risk-of-bias assessment or quantitative evidence synthesis was performed.

## 3. Overview of Endocrine-Disrupting Chemicals

Endocrine-disrupting chemicals are exogenous agents capable of altering physiological homeostasis through disruption of hormonal signaling and endocrine regulation [6]. These compounds are widely present across diverse environmental sources, including food products, plastics, pesticides, industrial compounds, and personal care products, leading to human exposure through oral, inhalational, and dermal routes. Owing to their ability to exert biological activity even at low doses and to induce persistent effects during critical developmental windows, EDCs are increasingly recognized as a major public health concern [4,5].

Early research on endocrine-disrupting chemicals (EDCs) focused predominantly on the reproductive and thyroid systems, whereas the adrenal cortex received comparatively little attention [6,7]. Since the early 2000s, increasing experimental and epidemiological evidence has highlighted the adrenal gland as an important target of EDC exposure, particularly with respect to steroidogenesis and adrenocortical function [8,9]. In particular, the prolonged exposure of a large population in the Veneto region of Italy to PFAS-contaminated drinking water between 1980 and 2013 has provided an important natural experiment for evaluating the health effects of environmental EDC exposure. This event further highlighted the remarkable environmental persistence and bioaccumulative potential of PFASs, demonstrating their ability to accumulate extensively in humans [10].

In recent years, there has been a marked increase in studies investigating the relationship between EDC exposure and hypertension [11]. In a study conducted by Bae and colleagues, bisphenol A (BPA) exposure was found to be associated with elevated blood pressure, and the estrogenic activity of BPA was proposed to contribute to its cardiovascular effects [12]. Estrogen receptors (ERα and ERβ) are widely expressed throughout the cardiovascular system and play key roles in regulating endothelial function, vascular tone, nitric oxide bioavailability, and blood pressure homeostasis [13,14].

## 4. Cardiovascular Effects of Endocrine-Disrupting Chemicals

The vascular endothelium plays a central role in the maintenance of vascular tone, inflammatory responses, and hemostatic balance [15]. Endothelial dysfunction is characterized by reduced nitric oxide bioavailability, increased oxidative stress, and inflammatory activation [16,17]. Under physiological conditions, the ACE2/Ang-(1–7)/MasR axis preserves vascular homeostasis through vasodilatory, anti-inflammatory, and antioxidative effects, whereas Ang II/AT1R-mediated signaling disrupts this balance, promoting vascular injury and remodeling [18,19,20]. This process is considered one of the key mechanisms underlying the development of cardiovascular disorders, including hypertension, atherosclerosis, myocardial infarction, stroke, and peripheral arterial disease [21]. Environmental toxicants, particularly heavy metals, have long been recognized as important contributors to cardiovascular disease and hypertension. Current evidence indicates that these effects are mediated through multiple interconnected mechanisms [22,23]. Heavy metals represent important environmental risk factors that should not be overlooked in the context of cardiovascular disease, and cadmium and lead in particular have been implicated in hypertension development because of their adverse effects on vascular structure and function [24]. Studies evaluating cadmium exposure have further demonstrated differences in renal cadmium accumulation between normotensive and hypertensive individuals [25,26].

Beyond heavy metals, growing evidence suggests that multiple endocrine-disrupting chemicals act through common mechanisms, including oxidative stress, endothelial dysfunction, inflammation, and altered hormonal signaling [11]. Among these compounds, bisphenol A (BPA) is one of the most extensively investigated endocrine-disrupting chemicals with regard to cardiovascular effects. BPA has been shown to modulate both genomic and non-genomic signaling pathways through ERα, ERβ, and GPER1/GPR30 [11,27].

At the molecular level, the vascular effects of estrogenic EDCs may involve both genomic and rapid non-genomic signaling. Genomic signaling is primarily mediated by ligand-dependent activation of nuclear estrogen receptors, particularly ERα and ERβ, which subsequently regulate transcription of genes involved in endothelial function, vascular tone, inflammation, and cellular proliferation. In contrast, non-genomic signaling may occur through membrane-associated estrogen receptors and GPER1/GPR30, leading to rapid activation of intracellular pathways such as PI3K/Akt, MAPK, calcium-dependent signaling, and eNOS-related nitric oxide production. These pathways may interact with oxidative stress, inflammatory signaling, and vascular smooth muscle cell responses, thereby influencing endothelial function and vascular remodeling. Importantly, these molecular effects do not occur in isolation but may intersect with conventional cardiovascular risk pathways, including obesity, insulin resistance, smoking, high dietary sodium intake, and aging, which themselves promote oxidative stress, endothelial dysfunction, inflammation, and altered vascular reactivity. This overlap may provide a biological basis for additive or modifying effects of EDC exposure on established hypertension risk pathways [11,13,14,27]. Recent evidence further highlights GPER as an important regulator of cardiovascular physiology and disease, with emerging roles in vascular tone, endothelial function, inflammation, and hypertension [28].

Although acute exposure may induce vasodilation by increasing nitric oxide production, chronic exposure has been associated with endothelial dysfunction mediated by enhanced oxidative stress and reduced nitric oxide bioavailability [29]. Increased serum nitric oxide levels already reflect early oxidative stress and endothelial alterations [30]. In addition, BPA is thought to influence cardiovascular programming during early life through neurohormonal and epigenetic mechanisms [31]. Collectively, these findings suggest that BPA may exert divergent effects on vascular homeostasis depending on the duration of exposure, with potentially protective effects in the short term but detrimental consequences following prolonged exposure [11]. In addition, SIRT6 has emerged as a potential protective regulator of cardiovascular homeostasis through modulation of inflammatory signaling, oxidative stress, endothelial repair, and vascular injury, although its specific role in EDC-related hypertension remains to be established [32].

Impairment of endogenous vascular repair may represent an additional mechanism contributing to EDC-related vascular dysfunction. Endothelial progenitor cells participate in endothelial maintenance and repair through proliferative, migratory, and angiogenic functions, whereas oxidative stress and inflammation may impair their reparative capacity. Endothelial injury is also associated with the release of endothelial microparticles and extracellular vesicles, which may influence nitric oxide signaling, inflammation, coagulation, and vascular remodeling. Thus, disruption of the balance between endothelial injury and repair could represent a complementary mechanism linking EDC exposure to hypertension, although direct evidence for EDC-specific effects on these pathways remains limited [15,16,17,21,33].

## 5. EDCs and the Pathophysiology of Hypertension

**Figure 1:**
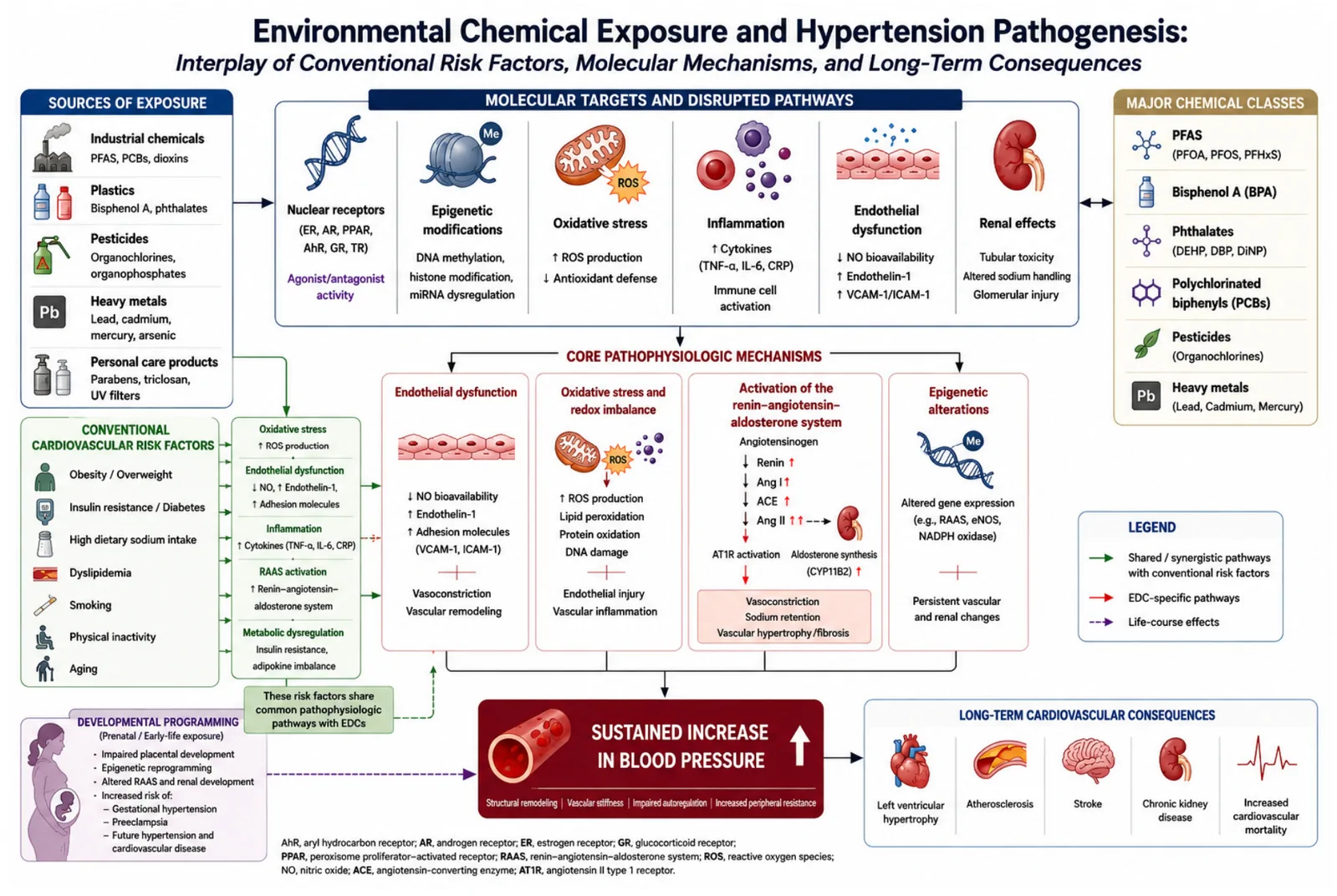
Proposed mechanisms linking environmental chemical exposure and conventional cardiovascular risk factors to hypertension pathogenesis. Environmental chemicals, including PFASs, bisphenol A, phthalates, polychlorinated biphenyls, pesticides, and heavy metals, may influence blood pressure through interconnected molecular and pathophysiological pathways involving nuclear receptor signaling, epigenetic modifications, oxidative stress, inflammation, endothelial dysfunction, renal injury, and activation of the renin–angiotensin–aldosterone system. These mechanisms may overlap with pathways associated with conventional cardiovascular risk factors, including obesity, insulin resistance, high dietary sodium intake, smoking, physical inactivity, dyslipidemia, and aging, thereby potentially amplifying vascular and renal dysfunction. Prenatal and early-life exposure may additionally promote developmental programming, increasing susceptibility to hypertensive disorders of pregnancy, future hypertension, and long-term cardiovascular disease.

In its Second Scientific Statement on endocrine-disrupting chemicals, the Endocrine Society emphasized the potential role of EDCs in the development of chronic diseases through disruption of multiple biological pathways. EDCs can interfere with hormone synthesis, secretion, transport, metabolism, receptor binding, and downstream signaling, while exposure during critical developmental windows may result in persistent physiological effects through epigenetic and developmental mechanisms. In the context of hypertension, accumulating experimental and epidemiological evidence suggests that EDC exposure may be associated with several interconnected mechanisms, including oxidative stress, endothelial dysfunction, inflammatory activation, vascular stiffness, renal injury, and activation of the renin–angiotensin–aldosterone system [34] (Figure 1).

These mechanisms should not be viewed as independent pathways but rather as components of an interconnected pathophysiological network. Oxidative stress and inflammatory signaling may impair endothelial function, whereas renal injury and RAAS/mineralocorticoid receptor activation promote sodium retention, vascular remodeling, and increased vascular resistance. Their convergence may therefore facilitate the transition from transient molecular disturbances to sustained elevations in blood pressure.

Environmental chemicals, including PFASs, bisphenol A, phthalates, polychlorinated biphenyls, pesticides, and heavy metals, may contribute to hypertension through multiple interconnected mechanisms involving oxidative stress, endothelial dysfunction, inflammation, epigenetic modifications, renal injury, and activation of the renin–angiotensin–aldosterone system. Prenatal exposure may additionally promote developmental programming, thereby increasing susceptibility to hypertensive disorders of pregnancy and long-term cardiovascular disease.

In recent years, endocrine-disrupting chemicals, particularly estrogenic compounds such as bisphenol A (BPA) and certain polychlorinated biphenyls (PCBs), as well as phthalates and diethylstilbestrol (DES), have been implicated in vascular remodeling through mechanisms involving oxidative stress, inflammation, vascular smooth muscle cell proliferation, and extracellular matrix remodeling [35,36]. In particular, PCB153 and BPA have been reported to promote reactive oxygen species (ROS) generation, which may contribute to endothelial dysfunction, arterial stiffness, and vascular inflammation, providing potential mechanistic links to hypertension and other cardiovascular diseases [37,38,39]. Exposure to EDCs during critical developmental periods has been shown to influence organ development and physiological function through epigenetic mechanisms [40,41]. Although findings regarding the association between BPA exposure and cardiovascular risk remain inconsistent, experimental and epidemiological evidence suggests potential adverse effects of BPA on cardiovascular function, including endothelial dysfunction and alterations in blood pressure regulation [42]. Moreover, prenatal phthalate exposure has been linked to increased systolic blood pressure in adulthood, particularly among males [43].

Various EDCs, including per- and polyfluoroalkyl substances (PFASs) and polychlorinated biphenyl 126 (PCB126), have been shown to stimulate aldosterone biosynthesis through upregulation of CYP11B2 expression, thereby promoting excess aldosterone production, a biologically plausible mechanism potentially linking these exposures to hypertension. Experimental evidence suggests that EDC-induced aldosterone biosynthesis is mediated, at least in part, by oxidative stress-dependent signaling pathways. Aldosterone represents a biologically plausible link between environmental endocrine disruption and blood pressure regulation. Increased aldosterone production promotes mineralocorticoid receptor activation in the kidney, enhancing sodium reabsorption and extracellular volume expansion and thereby contributing to sustained elevations in blood pressure. However, the cardiovascular effects of aldosterone extend beyond its renal actions. Excessive mineralocorticoid receptor signaling may promote oxidative stress, endothelial dysfunction, vascular inflammation, fibrosis, and vascular remodeling, mechanisms that can further increase vascular resistance and amplify hypertensive cardiovascular injury. In this context, EDC-induced alterations in adrenal steroidogenesis may provide a mechanistic bridge between environmental exposure and established pathways involved in hypertension. The observation that PFAS-induced CYP11B2 expression can be attenuated by inhibition of reactive oxygen species further supports an interaction between oxidative stress and adrenal steroidogenesis. Taken together, these findings raise the possibility that chronic exposure to selected EDCs may contribute to excess aldosterone production and subsequently reinforce both renal and vascular mechanisms involved in hypertension [44,45,46]. A recent experimental study demonstrated that phthalates and their alternatives can disrupt adrenal steroidogenesis in vitro, particularly through alterations in CYP11B1- and CYP11B2-mediated cortisol and aldosterone biosynthesis [47]. In addition, newer “phthalate-free” plasticizers such as DEHT and DINCH may exert similar endocrine effects, reinforcing the concept of “regrettable substitution,” whereby replacement chemicals may retain or even reproduce the biological effects of the compounds they were intended to replace [47,48].

These mechanistic observations are supported by epidemiological evidence linking PFAS exposure to hypertension. A recent meta-analysis by Pan and colleagues including 81,096 participants evaluated the association between multiple PFAS subtypes and hypertension. Exposure to PFOS, PFOA, and PFNA was associated with a higher risk of hypertension, with stronger associations observed in men than in women [49]. Beyond PFAS- and plastic-associated EDCs, recent epidemiological evidence has also linked herbicide exposure to hypertension risk. In a nested case–control study conducted in Henan, China, herbicide exposure was positively associated with hypertension prevalence, while exposure to herbicide mixtures was shown to further increase hypertension risk. Mechanistic analyses suggested that this relationship may be mediated, at least in part, through biomarkers of cellular senescence [50].

The epidemiological evidence should nevertheless be interpreted in the context of substantial heterogeneity across studies. Reported associations may vary according to the specific chemical or metabolite assessed, exposure level and timing, population characteristics, sex, and methods used for exposure and blood pressure assessment. This is particularly relevant for nonpersistent EDCs, for which single biomarker measurements may not adequately reflect long-term exposure. In addition, differences in co-exposures, background cardiovascular risk, and study design may contribute to inconsistent findings [42,43,49]. Importantly, real-world exposure rarely occurs for a single chemical in isolation, and cumulative or interactive effects of multiple EDCs may not be adequately captured by single-chemical analyses [51]. Accordingly, current evidence supports associations between selected EDC exposures and hypertension but may underestimate the cardiovascular consequences of complex environmental mixtures.

Nevertheless, despite the growing body of mechanistic and epidemiological evidence suggesting an association between EDC exposure and hypertension, the translation of these findings into clinical hypertension guidelines remains limited owing to the restricted ability of human studies to establish causality and the substantial heterogeneity in exposure assessment.

## 6. Endocrine-Disrupting Chemicals in Hypertensive Disorders of Pregnancy

Pregnancy represents a critical period characterized by heightened susceptibility to environmental exposures, particularly endocrine-disrupting chemicals (EDCs). Current evidence suggests that EDC exposure may be associated with a range of adverse pregnancy outcomes, particularly gestational hypertension and preeclampsia. In addition, prenatal EDC exposure is thought to contribute to long-term cardiometabolic consequences through epigenetic modifications and transgenerational effects [52]. Beyond their cardiovascular effects, these compounds have also been associated with adverse reproductive outcomes, including infertility, implantation failure, spontaneous abortion, and preterm birth, as well as hypertensive disorders of pregnancy [53].

Pregnancy is accompanied by profound cardiovascular, endocrine, renal, and placental adaptations that are required to maintain adequate maternal perfusion and fetal growth. These physiological changes may also increase susceptibility to environmental endocrine disruption. Normal pregnancy is characterized by substantial alterations in vascular resistance, plasma volume, renal sodium regulation, and hormonal regulation of blood pressure, while successful placentation requires tightly coordinated trophoblast invasion, spiral artery remodeling, and angiogenic signaling. Interference with these processes during critical stages of gestation may therefore have disproportionate effects on maternal vascular homeostasis [54].

EDCs may influence this balance through several complementary pathways, including oxidative stress, endothelial dysfunction, altered steroid hormone signaling, disruption of placental angiogenesis, and changes in the renin–angiotensin–aldosterone system. The timing of exposure may be particularly important, as disruption of placental development during critical stages of gestation may predispose to later hypertensive complications. These observations provide a biological rationale for evaluating EDC exposure not only in relation to maternal blood pressure during pregnancy, but also in the context of placental function and long-term cardiovascular health in both mothers and offspring [55,56].

Human epidemiological studies have reported associations between selected EDC exposures and gestational blood pressure or hypertensive disorders of pregnancy, although the findings remain heterogeneous [55,56,57]. In contrast, many of the proposed biological mechanisms linking EDC exposure to preeclampsia, including alterations in placental function, oxidative stress, endothelial dysfunction, and epigenetic regulation, are derived predominantly from experimental and mechanistic evidence [54,58,59].

Meta-analyses have demonstrated that exposure to PFOA, PFOS, and PFNA is associated with an increased risk of preeclampsia, whereas exposure to PFOA and PFHxS has been associated with gestational hypertension. Among phthalates, only monoethyl phthalate (MEP) was found to be associated with gestational hypertension, while no significant association was identified for BPA [56]. In a recent cohort study, exposure to DiNP and DEHTP during early pregnancy was associated with increased gestational blood pressure and hypertensive disorders of pregnancy. These findings suggest that early pregnancy represents a particularly vulnerable developmental window for EDC exposure, potentially through mechanisms involving impaired placental vascularization and activation of the renin–angiotensin–aldosterone system [58].

Experimental and mechanistic evidence suggests that EDCs may influence pathways relevant to preeclampsia through epigenetic mechanisms. In particular, BPA, phthalates, and PFAS compounds have been proposed to disrupt VEGF-mediated angiogenesis through alterations in microRNA expression, including miR-210 and miR-126. These compounds may also contribute to sFlt-1/PlGF imbalance, thereby promoting placental dysfunction, oxidative stress, and endothelial injury. Furthermore, EDC-induced alterations in DNA methylation have been suggested to amplify inflammatory responses and contribute to the pathophysiology of preeclampsia [57].

Evidence regarding the association between PFAS exposure and hypertensive disorders of pregnancy remains heterogeneous. In a large cohort study conducted in Ronneby, Sweden, involving high levels of PFOS and PFHxS exposure, no significant increase in the risk of gestational hypertension or preeclampsia was observed. Nevertheless, PFAS exposure has been proposed to influence pregnancy-related complications through mechanisms involving placental vascularization, trophoblast function, oxidative stress, and epigenetic alterations [59]. A recent review evaluating epidemiological and experimental studies investigating the relationship between EDC exposure and preeclampsia concluded that both in vivo and in vitro evidence supports biologically plausible links between EDC exposure and pathways involved in preeclampsia, including impaired trophoblast invasion, defective spiral artery remodeling, oxidative stress, and endothelial dysfunction. The review also highlighted PFASs as the most extensively investigated class of EDCs in relation to preeclampsia [60].

The major EDC classes, their dominant biological mechanisms, vascular and renal effects, and potential clinical implications in hypertension are summarized in Table 1.

In a review by Kahn and colleagues, environmental toxicants were proposed to be associated not only with hypertensive disorders during pregnancy but also with long-term maternal cardiovascular health. Women with a history of preeclampsia were reported to have an increased risk of future cardiovascular disease, stroke, heart failure, and chronic hypertension later in life [29].

## 7. Clinical Implications and Public Health Perspectives

Accumulating evidence linking EDC exposure to hypertension raises important clinical and public health considerations. Although traditional cardiovascular risk factors remain central to hypertension prevention and management, environmental chemical exposure may represent an additional and potentially modifiable component of cardiovascular risk [61]. This perspective may be particularly relevant for populations with greater susceptibility or higher exposure, including pregnant women, individuals with occupational exposure to environmental chemicals, and patients with established cardiometabolic risk factors. In clinical practice, obtaining information on occupational and environmental exposures may therefore complement conventional risk assessment in selected patients, particularly when substantial or persistent exposure is suspected [62]. Accordingly, a focused occupational and environmental exposure history may be reasonable in selected patients with suspected substantial or persistent exposure, while routine EDC biomonitoring cannot currently be recommended for hypertension risk stratification.

Nevertheless, no validated role for routine EDC biomonitoring in hypertension risk stratification or management has been established. Biomonitoring remains challenging because exposure patterns vary considerably between compounds, some EDCs have short biological half-lives, and single measurements may not accurately reflect long-term exposure. Moreover, individuals are exposed to complex mixtures of chemicals rather than isolated compounds, making it difficult to attribute cardiovascular effects to a single exposure [51]. These limitations, together with the predominance of observational evidence and substantial heterogeneity in exposure assessment, currently limit the translation of EDC-related findings into routine hypertension risk assessment and management.

From a preventive perspective, reducing avoidable exposure may nevertheless be reasonable, particularly in susceptible populations. Practical approaches may include limiting unnecessary contact with known sources of EDCs, reducing the use of plastics for food storage and heating, minimizing consumption of highly packaged foods, and following appropriate occupational protection measures [62,63]. However, the burden of exposure reduction should not be placed solely on individuals, as many EDCs are widespread in consumer products, food systems, workplaces, and the broader environment. Population-level strategies, including improved chemical surveillance, safer product formulation, occupational exposure control, and regulatory policies that consider endocrine activity and cardiovascular outcomes, are therefore likely to be important components of prevention [63].

An additional challenge is that chemical regulation has traditionally focused on individual compounds, whereas real-world exposure involves multiple chemicals with potentially additive, synergistic, or interacting effects [51]. Greater consideration of cumulative and mixture exposures may therefore improve both environmental risk assessment and regulatory decision-making. At present, EDC exposure may be more appropriately regarded as an emerging environmental determinant of hypertension rather than an established clinical risk factor requiring routine testing. Further prospective and interventional evidence will be necessary to determine whether systematic exposure assessment or exposure-reduction strategies can meaningfully improve blood pressure control and reduce cardiovascular events.

## 8. Artificial Intelligence and Environmental Health Research

Machine learning has emerged as an increasingly valuable analytical approach in environmental health research owing to its ability to process and analyze large, complex datasets. In a study evaluating eight different machine learning algorithms, environmental chemical exposures were identified as important predictors of hypertension, demonstrating the potential of machine learning-based models to characterize complex relationships between environmental exposures and blood pressure outcomes [37].

The application of artificial intelligence may be particularly relevant to EDC research because real-world exposure rarely occurs to a single chemical in isolation. Instead, individuals are simultaneously exposed to multiple environmental chemicals whose effects may be nonlinear, correlated, or interactive. Machine learning approaches can facilitate the analysis of such high-dimensional exposure patterns and may identify exposure patterns associated with cardiovascular risk [64]. Integration of environmental exposure data with demographic characteristics, clinical variables, and biological markers may further support a more comprehensive approach to hypertension risk assessment. In this context, AI-based exposomic approaches may be particularly useful for integrating multiple simultaneous chemical exposures and identifying complex exposure interactions that may not be adequately captured by conventional single-chemical analyses. Such approaches may therefore provide a methodological bridge between real-world environmental mixtures and their potential contribution to hypertension risk.

Beyond exposure characterization, AI-based approaches may have potential applications in risk stratification and prevention. By integrating environmental exposure profiles with conventional cardiovascular risk factors, demographic characteristics, and biological markers, machine-learning models may help identify individuals or subgroups with increased susceptibility to EDC-related blood pressure alterations. Such approaches could eventually support targeted preventive strategies, including prioritization of occupational or environmental exposure assessment and individualized exposure-reduction counseling. Longitudinal AI models may also help characterize changes in exposure patterns and blood pressure risk over time. However, these applications remain investigational, and prospective validation is required before AI-derived environmental risk estimates can be incorporated into routine hypertension prevention or management.

Despite these opportunities, the clinical application of artificial intelligence in environmental hypertension research remains at an early stage. Model performance may be affected by exposure measurement error, differences in study populations, incomplete characterization of chemical mixtures, and limited external validation. Furthermore, the complexity and limited interpretability of some machine learning models may restrict their ability to provide biologically meaningful or clinically actionable information [64,65,66].

Future studies should therefore prioritize standardized exposure assessment, transparent and interpretable modeling approaches, external validation across diverse populations, and prospective evaluation of AI-derived risk estimates. With these advances, artificial intelligence may ultimately complement conventional epidemiological methods by improving the characterization of complex environmental exposures and their contribution to hypertension risk.

## 9. Future Directions and Research Priorities

Despite growing evidence linking EDC exposure to hypertension, several important knowledge gaps remain. Much of the available human evidence is derived from cross-sectional or observational studies, limiting the ability to establish temporal and causal relationships. Future research should therefore prioritize large prospective cohorts with repeated measurements of both environmental exposures and blood pressure over time [67]. Repeated biomonitoring may be particularly important for nonpersistent EDCs with short biological half-lives, for which a single measurement may not adequately represent long-term exposure [68]. Harmonization of exposure assessment, outcome definitions, and analytical approaches across studies would also improve comparability and facilitate more reliable evidence synthesis.

Another important priority is to move beyond the traditional single-chemical approach. Human populations are exposed to complex mixtures of EDCs throughout the life course, and the cardiovascular effects of these exposures may depend on their timing, duration, and co-occurrence [64]. Future studies should therefore characterize cumulative and mixture exposures and investigate potentially vulnerable periods, including fetal development, pregnancy, childhood, and adolescence. Greater attention should also be given to sex-specific effects, as differences in endocrine signaling, metabolism, and exposure patterns may modify susceptibility to EDC-related changes in blood pressure [69]. Integration of mechanistic studies with prospective epidemiological data will be essential to clarify whether observed associations reflect causal pathways and to identify biological processes that may be suitable for targeted prevention. Importantly, future research should extend beyond observational associations to determine whether exposure reduction translates into measurable cardiovascular benefit. Existing intervention studies indicate that dietary and personal-care-product interventions can reduce exposure biomarkers for selected EDCs, but whether such reductions lead to improvements in blood pressure or other cardiovascular outcomes remains uncertain [70].

Future intervention studies should therefore evaluate whether individual- or population-level exposure-reduction strategies influence blood pressure, intermediate vascular outcomes, or incident hypertension. Advances in exposomics, high-throughput biomonitoring, and artificial intelligence may further facilitate the integration of complex environmental, clinical, and biological data and improve the identification of exposure patterns associated with hypertension. Ultimately, multidisciplinary collaboration among clinicians, epidemiologists, toxicologists, environmental scientists, data scientists, and policymakers will be necessary to translate emerging evidence on EDCs into effective strategies for hypertension prevention.

## 10. Conclusions

A growing body of experimental and epidemiological evidence suggests an association between EDC exposure and hypertension, supported by several biologically plausible mechanisms. Endothelial dysfunction, oxidative stress, inflammation, epigenetic alterations, and activation of the renin–angiotensin–aldosterone system appear to represent the principal biological mechanisms underlying this association. In addition, prenatal exposure to EDCs may be linked to hypertensive disorders of pregnancy, including gestational hypertension and preeclampsia, and may contribute to adverse long-term cardiovascular outcomes. Nevertheless, the interpretation of current evidence remains limited by the restricted ability of human studies to establish causality and by substantial heterogeneity in exposure assessment. Future research should prioritize prospective studies with standardized and repeated exposure assessment, improved characterization of chemical mixtures, and intervention studies to determine whether reducing EDC exposure translates into meaningful improvements in blood pressure and cardiovascular outcomes.

## Figures and Tables

**Table 1:** Translational and clinical implications of endocrine-disrupting chemical exposure in hypertension. Major classes of endocrine-disrupting chemicals, their dominant biological mechanisms, vascular and renal effects, and potential clinical implications in hypertension and hypertensive disorders of pregnancy are summarized. Shared mechanistic pathways include oxidative stress, endothelial dysfunction, inflammation, altered steroidogenesis, epigenetic modifications, and activation of the renin–angiotensin–aldosterone system.

EDC Class	Representative Compounds	Dominant Biological Mechanisms	Major Vascular/ Renal Effects	Clinical Implications
**PFASs**	PFOS, PFOA, PFNA	CYP11B2 upregulation, RAAS activation, oxidative stress	Aldosterone excess, endothelial dysfunction, renal sodium retention	Hypertension, gestational hypertension, preeclampsia
**Bisphenols**	BPA	Estrogen receptor signaling, ROS generation, epigenetic alterations	Endothelial dysfunction, vascular remodeling	Elevated blood pressure, arterial stiffness
**Phthalates**	DEHP, DiNP, MEP	Oxidative stress, steroidogenesis dysregulation, inflammation	Reduced vascular elasticity, altered adrenal steroidogenesis	Hypertension, increased systolic BP, hypertensive pregnancy disorders
**PCBs**	PCB126, PCB153	ROS production, inflammatory activation	Arterial stiffness, endothelial injury	Hypertension, cardiovascular disease
**Heavy metals**	Cadmium, lead	Endothelial toxicity, oxidative injury	Vascular dysfunction, renal injury	Hypertension, CKD, atherosclerosis
**Pesticides/Herbicides**	Organochlorines, herbicide mixtures	Cellular senescence, inflammatory signaling	Vascular inflammation	Increased hypertension risk

## Data Availability

No new data were created or analyzed in this study. Data sharing is not applicable to this article.
